# Mutation of Androgen Receptor N-Terminal Phosphorylation Site Tyr-267 Leads to Inhibition of Nuclear Translocation and DNA Binding

**DOI:** 10.1371/journal.pone.0126270

**Published:** 2015-05-07

**Authors:** Mehmet Karaca, Yuanbo Liu, Zhentao Zhang, Dinuka De Silva, Joel S. Parker, H. Shelton Earp, Young E. Whang

**Affiliations:** 1 Department of Pathology and Laboratory Medicine, University of North Carolina at Chapel Hill, Chapel Hill, NC, 27599, United States of America; 2 Lineberger Comprehensive Cancer Center, University of North Carolina at Chapel Hill, Chapel Hill, NC, 27599, United States of America; 3 Department of Hematology, Beijing Tiantan Hospital, Capital Medical University, Beijing 100050, China; 4 Department of Genetics, University of North Carolina at Chapel Hill, Chapel Hill, NC 27599, United States of America; 5 Department of Medicine, University of North Carolina at Chapel Hill, Chapel Hill, NC 27599, United States of America; 6 Department of Pharmacology, University of North Carolina at Chapel Hill, Chapel Hill, NC 27599, United States of America; Baylor College of Medicine, UNITED STATES

## Abstract

Reactivation of androgen receptor (AR) may drive recurrent prostate cancer in castrate patients. Ack1 tyrosine kinase is overexpressed in prostate cancer and promotes castrate resistant xenograft tumor growth and enhances androgen target gene expression and AR recruitment to enhancers. Ack1 phosphorylates AR at Tyr-267 and possibly Tyr-363, both in the N-terminal transactivation domain. In this study, the role of these phosphorylation sites was investigated by characterizing the phosphorylation site mutants in the context of full length and truncated AR lacking the ligand-binding domain. Y267F and Y363F mutants showed decreased transactivation of reporters. Expression of wild type full length and truncated AR in LNCaP cells increased cell proliferation in androgen-depleted conditions and increased colony formation. However, the Y267F mutant of full length and truncated AR was defective in stimulating cell proliferation. The Y363F mutant was less severely affected than the Y267F mutant. The full length AR Y267F mutant was defective in nuclear translocation induced by androgen or Ack1 kinase. The truncated AR was constitutively localized to the nucleus. Chromatin immunoprecipitation analysis showed that it was recruited to the target enhancers without androgen. The truncated Y267F AR mutant did not exhibit constitutive nuclear localization and androgen enhancer binding activity. These results support the concept that phosphorylation of Tyr-267, and to a lesser extent Tyr-363, is required for AR nuclear translocation and recruitment and DNA binding and provide a rationale for development of novel approaches to inhibit AR activity.

## Introduction

Prostate cancer remains the second leading cause of cancer death among American men due to the progression after androgen deprivation therapy of initially hormone dependent prostate cancer. Current evidence indicates that reactivation of androgen receptor (AR) in tumor cells may play a critical role in the development of castration resistant prostate cancer (CRPC) [1]. Multiple mechanisms of AR activation in CRPC tumor cells have been characterized. These include increased expression of AR mRNA, AR gene amplification, and point mutations in AR [2–5]. Modest overexpression of AR in prostate cancer cells was sufficient to promote the castrate resistant growth of xenograft tumors [2]. Furthermore, increased expression of coactivators such as SRC1 or TIF2/SRC2/NCOA2 may enhance AR transactivation [6]. Intratumoral de novo biosynthesis of androgen in CRPC tumor cells may also provide for ligand-dependent activation of AR [3,7,8]. Splice variants of AR lacking the ligand-binding domain have been found to be expressed in prostate cancer cells [9–12]. Emergence of constitutively active AR variants may mediate growth and progression of prostate cancer in the castrate host and may confer resistance to new potent antiandrogens [13,14].

In addition, activation of AR through crosstalk with kinase signaling pathways has been postulated as a potential mechanism for recurrent tumor growth in the castrate environment [1]. AR protein undergoes phosphorylation at serine/threonine and tyrosine residues [15]. Guo et al demonstrated that Src kinase-mediated phosphorylation of AR at Tyr-534 induced nuclear location, recruitment of AR to the chromatin, and tumor growth in castrated animals [16]. Etk/BMX tyrosine kinase phosphorylates AR at Tyr-534 [17]. Fer tyrosine kinase, acting downstream of interleukin-6, phosphorylates AR at Tyr-223 [18]. Ack1 (activated cdc42-associated kinase, also known as Tnk2) is a nonreceptor tyrosine kinase that is overexpressed in several different tumor types, including prostate, and enhances tumor growth and invasion and metastasis [19–22]. For example, ectopic expression of activated Ack1 in prostate cancer cells enhanced the ability of androgen-dependent prostate cancer cells to grow as xenograft tumors in castrated animals [23]. Ack1 promoted AR target gene expression and recruitment and binding of AR to the regulatory regions of target genes, coincident with AR tyrosine phosphorylation. AR Tyr-267 phosphorylation by Ack1 was initially identified by mass spectroscopy and subsequently confirmed by phospho-specific antibodies [23–25]. AR Tyr-363 as a major Ack1-dependent phosphorylation site was suggested only by mutational analysis [23]. Mutation of these two sites in AR inhibited Ack1-induced AR transactivation and DNA binding as well as tumor growth. However, the role of these phosphorylation sites in general AR function in prostate cancer cells that have not been transfected with activated Ack1 has not been well characterized. In this study, the phenotype of phosphorylation site mutants of full length AR and the constitutively active AR that lacks the ligand-binding domain was investigated.

## Materials and Methods

### Cell lines and reagents

LNCaP cells [26] was initially obtained from American Type Culture Collection (Manassas, VA, catalog number CRL-1740) and were authenticated by short tandem repeat analysis performed by the Tissue Culture Facility of the University of North Carolina at Chapel Hill at the conclusion of this study. LNCaP cells were grown in phenol red-free RPMI 1640 supplemented with 10% fetal bovine serum. COS-7 cells (ATCC, CRL-1651) were grown in DMEM supplemented with 10% fetal bovine serum. Epidermal growth factor (EGF) and Gas6 were purchased from R&D Systems (Minneapolis, MN). Heregulin was a gift from Genentech (South San Francisco, CA). Phospho-Tyr-267 specific polyclonal antibody against AR was previously described [24]. For immunoblotting and immunofluorescence of AR, a mouse monoclonal antibody against AR (F39.4.1, Biogenex, San Ramon, CA) was used. For immunoprecipitation of AR, a polyclonal antibody against AR (C-19, Santa Cruz Biotechnology, Dallas, TX) was used. A mouse monoclonal antibody against FLAG and FLAG-conjugated agarose beads (Sigma-Aldrich, St. Louis, MO) were used for immnuoblotting and immunoprecipitation, respectively. Rabbit polyclonal antibodies against Laminin A/C (H-110) and 14-3-3 β (C-20) (Santa Cruz) were used in immunoblotting of subcellular fractions. Immunoblotting data shown in figures are representative of at least three independent experiments. Donkey anti-mouse fluorescein isothiocyanate (FITC)-labeled secondary antibody (Jackson ImmunoResearch, West Grove, PA) and mounting media with DAPI were used for staining AR and nucleus, respectively.

### Plasmids and reporter assays

For construction of AR mutants, the QuikChange Site-Directed Mutagenesis Kit (Strategene, La Jolla, CA) was used according to the manufacturer’s protocol. A stop codon was inserted after amino acid 660 in the wild type AR plasmid to generate the truncated AR construct. All mutations were confirmed by sequencing. LNCaP cells were plated (1.5 X 10^5^ cells per well) in 12-well plates the day before transfection. 150 ng of the AR construct (wild type or mutant) and 150 ng of the AR-dependent reporter construct (MMTV- or ARR2-PB-luciferase) were co-transfected using Effectene (Qiagen, Valencia, CA). Next day, medium was replaced with phenol-red free, serum free media and incubated for 8 hours. Cells were then treated with 10 nM dihydrotestosterone (DHT) (Sigma-Aldrich) or vehicle in serum free media for 16 hrs. Luciferase activity was measured, as previously described [23,24].

### Cell proliferation and soft agar colony formation

The AR fragments were subcloned into the pMSCV-puro retroviral vector. AR constructs contain both HA and FLAG tags. LNCaP cells were engineered to stably express AR wt or mutant AR constructs via retroviral transduction and selection as described [22]. Cells were cultured in a 96 well plate for 72 hours in media with charcoal-stripped serum and indicated concentrations of DHT. The colorimetric dye WST-8 (Dojindo, Rockville, MD) was added and absorbance at 450 nm was measured. Soft agar colony formation assays of LNCaP cells stably expressing vector, AR wt, or mutant AR constructs were performed as described previously [24].

### Subcellular fractionation of protein

Subcellular fractionation of cells was performed by using NE-PER Nuclear and Cytoplasmic Extraction Reagent (Pierce Biotechnology, Rockford, IL), supplemented with protease inhibitors to cytoplasmic extraction reagent I (CER I) and nuclear extraction reagent (NER) as described in their protocol. The protein content of cytoplasmic and nuclear extracts was determined with the Bradford assay reagent (Bio-Rad, Hercules, CA) and an equal amount of protein was used in immunoblotting.

### Immunofluorescence

COS-7 cells were counted and 3x10^4^ cells were plated in each well of two well chamber slides (LabTek II System, Nalge Nunc International, Naperville, IL). Next day, cells were transiently transfected with AR (100 ng) or vector (100 ng) along with constitutively active Ack1 (100 ng) or vector (100 ng) expression constructs, using FuGENE 6 (Roche Applied Science, Indianapolis, IN) and incubated for overnight. Media was changed to serum free medium and cells were serum starved for 8 hrs. After serum starvation, cells were either treated with DHT (10 nM) or vehicle (1% ethanol) for 2 hours. Next, media was removed from the cells and cells were rinsed with cold phosphate buffered saline (PBS) once for 5 minutes. The fixative (4% paraformaldehyde) was added to the cells and incubated at room temperature for 15 minutes. At the end of the fixation, cells were washed with cold PBS twice, and the blocking solution (0.3% Triton X and 5% donkey serum) was added and incubated for 30 minutes at room temperature. At the end of the incubation, blocking solutions were removed from the wells except the blank samples. Primary antibody, mouse anti-androgen receptor (F39.4.1, Biogenex, San Ramon, CA) made in 1:100 dilution in the blocking solution, was added on the cells and incubated at room temperature for two hour. At the end of incubation, primary antibody was removed from the cells and the cells were washed three times with cold PBS (5 minutes each wash). Secondary antibody, FITC-conjugated donkey anti mouse antibody, was prepared in 1:250 dilution in the blocking solution and added to the cells. The cells were incubated in the dark at 37°C for one hour. The secondary antibody was removed and the cells were washed with cold PBS three times. At the end of the wash, cover slips were placed with the mounting media which includes 4',6-diamidino-2-phenylindole (DAPI) (Vector Laboratories, Burlingame, CA) for nuclear staining. After cover slips were mounted, slides were kept in the dark cold room until images were captured.

### Chromatin immunoprecipitation

Chromatin immunoprecipitation analysis for recruitment of FLAG-tagged AR protein to the androgen response element III enhancer of canonical AR target genes, prostate specific antigen (PSA) and kallikrein-related peptidase 2 (KLK2), was performed as previously described [23]. mRNA levels of PSA and KLK2 by quantitative RT-PCR was determined as previously described [23].

### Microarray gene expression analysis and AR pathway score

Total RNA from LNCaP cells expressing vector, TR-WT-AR, and TR-AR-Y267F growing in androgen deprived conditions were isolated by using RNeasy Mini kit (Qiagen Inc., Valencia, CA) according to the manufacturer’s instructions. Two biological replicates were used per each cell line. RNA integrity was evaluated using the RNA 6000 Nano LabChip kit and a Bioanalyzer (Agilent Technologies Inc., Santa Clara, CA). RNA samples from TR-AR-WT or TR-AR-Y267F were assayed versus vector control as reference sample. The samples were hybridized onto the Agilent Whole Human Genome Oligonucleotide Microarray (4X44K) according to the manufacturer’s protocol at the UNC Genomics Core Facility. The microarrays were scanned on an Agilent scanner and the raw data extracted and deposited into the UNC Microarray Database. Individual channel values were background corrected and lowess normalized. The resulting log ratios were median centered by gene to produce the values for further gene expression analysis. From microarray gene expression data, a score representing AR transcription pathway activity was calculated. The previously published androgen response signature was used [27]. This signature is composed of genes that increase or decrease in response to androgen. For each sample, a t-statistic between the normalized expression of the genes expected to increase versus those expected to decrease in response to androgen was calculated as previously described [28]. A positive score indicates increased activation of the AR transcription pathway while a negative score indicates decreased activation of the pathway.

### Statistical analysis

The two-group t-test was used for the pair-wise group comparisons of wt to Y267F and wt to Y363F. These t-test p values have been adjusted using the Bonferroni method to account for multiple testing. Statistical analyses were performed with SAS statistical software (Version 9.2, SAS Institute Inc., Cary, NC). p value <0.05 was considered statistically significant.

## Results

### Ligand-independent AR activation is impaired in phosphorylation site mutants of truncated AR

**Fig 1:**
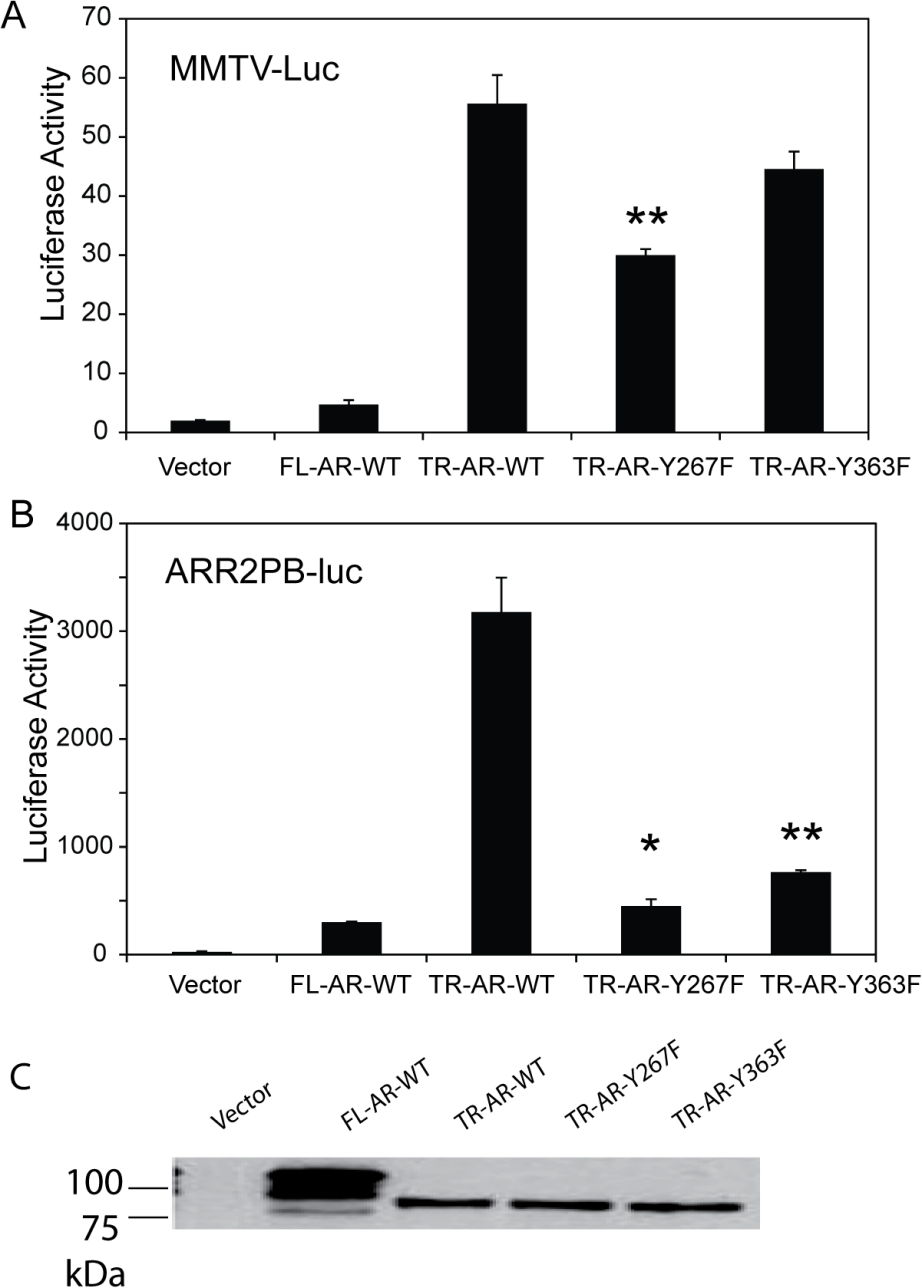
Phosphorylation site mutants of constitutively active truncated AR lacking the ligand-binding domain exhibit impaired transcriptional function in reporter assays. (A, B) LNCaP cells were transiently transfected with vector alone or the expression vector encoding full-length AR wt, truncated AR wt (TR-AR-WT), Y267F or Y363F mutants of truncated AR along with the AR-dependent reporter MMTV-luciferase (A) or ARR2-PB-luciferase (B). After 48 hours in androgen depleted media, luciferase activity was determined from cell lysates. Data shown with the mean + standard deviation of triplicate samples are representative of three independent experiments with similar results. *, p<0.001 vs TR-AR-WT; **, p<0.05 vs TR-AR-WT by two-group t-test adjusted using the Bonferroni method. (C) Cell lysates of COS-7 cells transiently transfected with respective AR constructs were immunoblotted with the monoclonal antibody against AR.

AR-dependent reporter assays showed impaired ligand-dependent and Ack1-induced activation of full length AR when Tyr-267 and Tyr-363 residues were mutated to phenylalanine [23]. To assess the role of these phosphorylation sites in ligand-independent AR activation, the phosphorylation site mutants Y267F and Y363F were tested in the context of truncated AR containing amino acid 1–660 (out of 919 amino acids of full length AR). This truncated AR protein retains the N-terminal transactivation domain and the central DNA binding domain. However, it is missing the C-terminal ligand-binding domain and is constitutively active in the absence of ligand [29]. The truncated AR bearing the Y267F mutation exhibited marked reduction in its ability to activate both MMTV and ARR2-PB reporters (Fig 1A and 1B). The Y363F mutant also showed decreased transcriptional activity, but to a less extent. Immunoblotting confirmed that protein levels of truncated AR withY267F and Y363F mutations were similar to that of wild type truncated AR (Fig 1C), although all three truncated AR proteins were expressed at lower levels than full length AR. Taken together, these results suggest that Tyr-267 and possibly Tyr-363 are essential for both ligand-dependent and-independent transcriptional activity of AR, and that loss of Tyr-267 exhibits more severe impairment of transcriptional function, compared to Tyr-363.

### Stably overexpressed wild type full length AR and truncated AR proteins are phosphorylated at Tyr-267 after growth factor treatment

**Fig 2:**
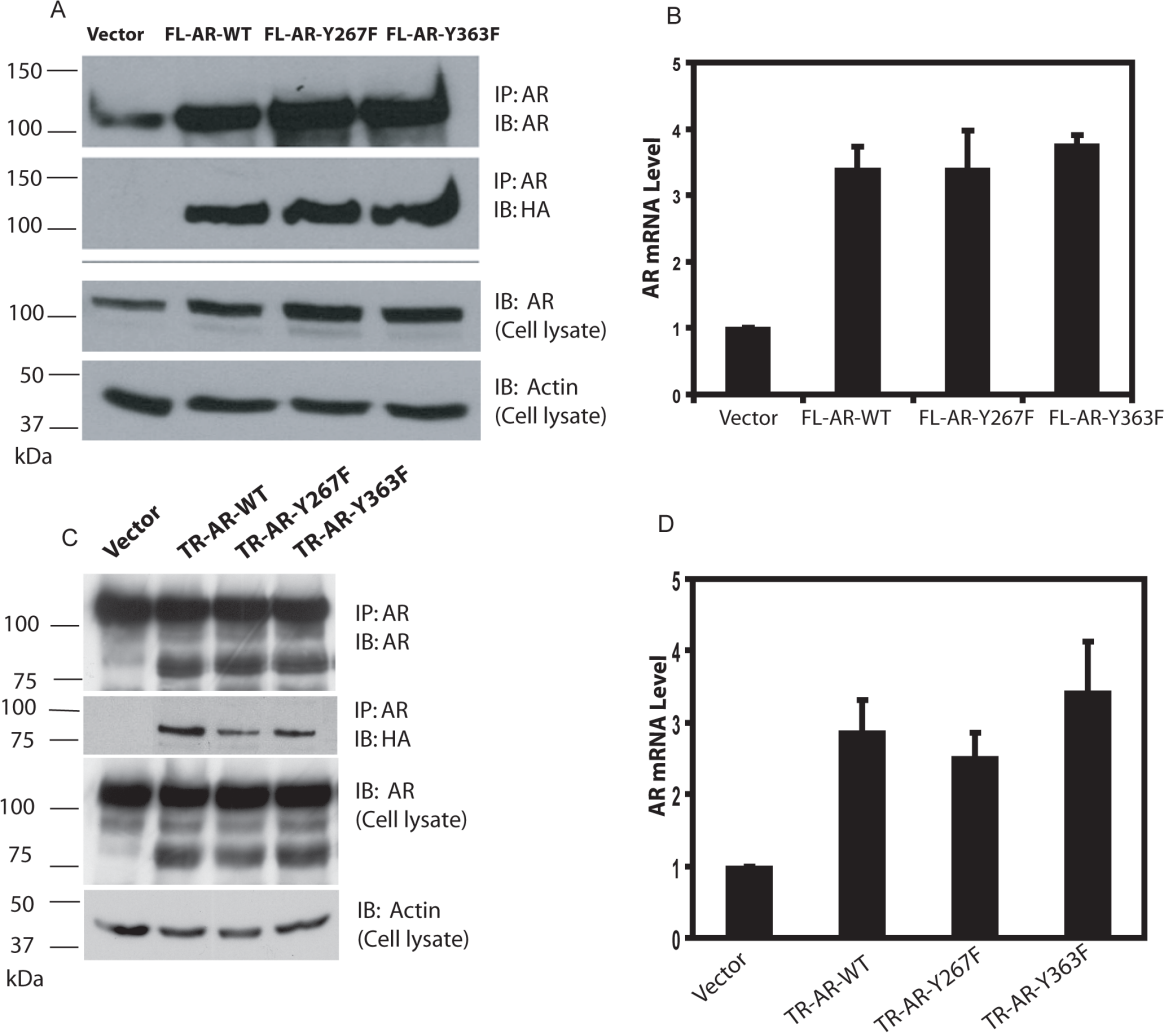
LNCaP cells stably overexpress full length or truncated AR or phosphorylation site mutants. LNCaP cells stably overexpressing AR constructs or vector control were derived by retrovirus-mediated transduction and antibiotic selection. (A) Lysates from LNCaP cells overexpressing full-length AR were subjected to immunoprecipitation with the AR antibody, followed by immunoblotting with the AR antibody or the epitope tag HA antibody, as indicated. Total cell lysates were also immunoblotted with the AR monoclonal antibody and actin antibody. (B) The mRNA levels of AR was determined by quantitative reverse transcription PCR of total RNA isolated from LNCaP cells stably overexpressing full-length AR. (C) Lysates from LNCaP cells stably overexpressing truncated AR were subjected to immunoprecipitation, followed by immunoblotting, as indicated. Total cell lysates were also immunoblotted with the AR monoclonal antibody and the actin antibody. (D) The mRNA levels of AR was determined by quantitative reverse transcription PCR of total RNA isolated from LNCaP cells stably overexpressing truncated AR.

**Fig 3:**
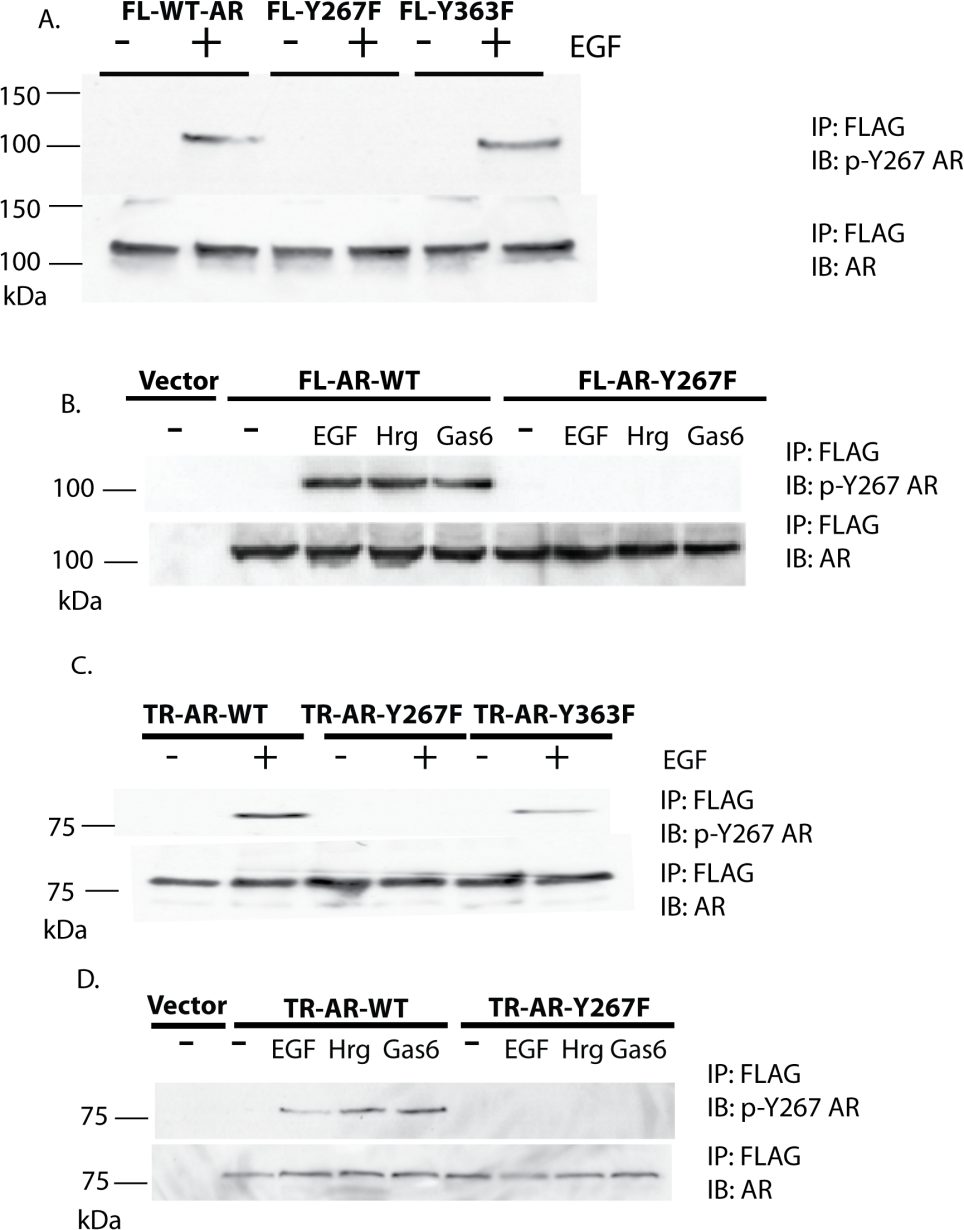
Exogenous AR stably expressed in LNCaP cells is phosphorylated at Tyr-267 after growth factor treatment. (A, B) LNCaP cells stably overexpressing full length AR were treated with EGF (100 ng/ml) or heregulin (10 ng/ml) or Gas6 (100 ng/ml) for 1 hr, as indicated. Cell lysates were subjected to immunoprecipitation with the FLAG antibody, followed by immunoblotting with the phospho-specific AR or total AR antibody. (C, D) LNCaP cells stably overexpressing truncated AR were treated with EGF (100 ng/ml) or heregulin (10 ng/ml) or Gas6 (100 ng/ml) for 1 hr, as indicated. Cell lysates were subjected to immunoprecipitation with the FLAG antibody, followed by immunoblotting with the phospho-specific AR or total AR antibody.

Overexpression of AR in androgen-dependent LNCaP cells enhances cell proliferation at suboptimal androgen concentrations [2,30]. The functional role of AR Tyr-267 and Tyr-363 sites in the cellular context was investigated by stable overexpression of wild type and mutant AR constructs. The total protein levels of full length AR in LNCaP cells stably expressing exogenous AR by retroviral transduction was 2–3 fold higher than endogenous AR in vector control cells (Fig 2A). Detection of exogenous AR by the epitope tag verified that wild type, Y267F, and Y363F AR were all expressed at comparable levels. In cells expressing truncated AR constructs, levels of wild type, Y267F, and Y363F AR migrating at about 80 kDa were similar (Fig 2C). AR mRNA levels in stably overexpressing cells were 3–4 folds higher than vector control cells in full length AR-expressing cells and 2–3 folds higher in truncated AR-expressing cells (Fig 2B and 2D). Immunoblotting with the phospho-Tyr-267 AR specific antibody demonstrated that both full length and truncated exogenous AR proteins were phosphorylated at Tyr-267 after stimulation with EGF, heregulin, or Gas6, as previously shown for endogenous AR protein [24] and that phosphorylation of Tyr-267 is not detected in Y267F AR protein (Fig 3). Mutation of Tyr-363 did not affect phosphorylation of Tyr-267.

### Phosphorylation site mutants of AR are defective in stimulating cell proliferation and anchorage-independent soft agar colony growth

**Fig 4:**
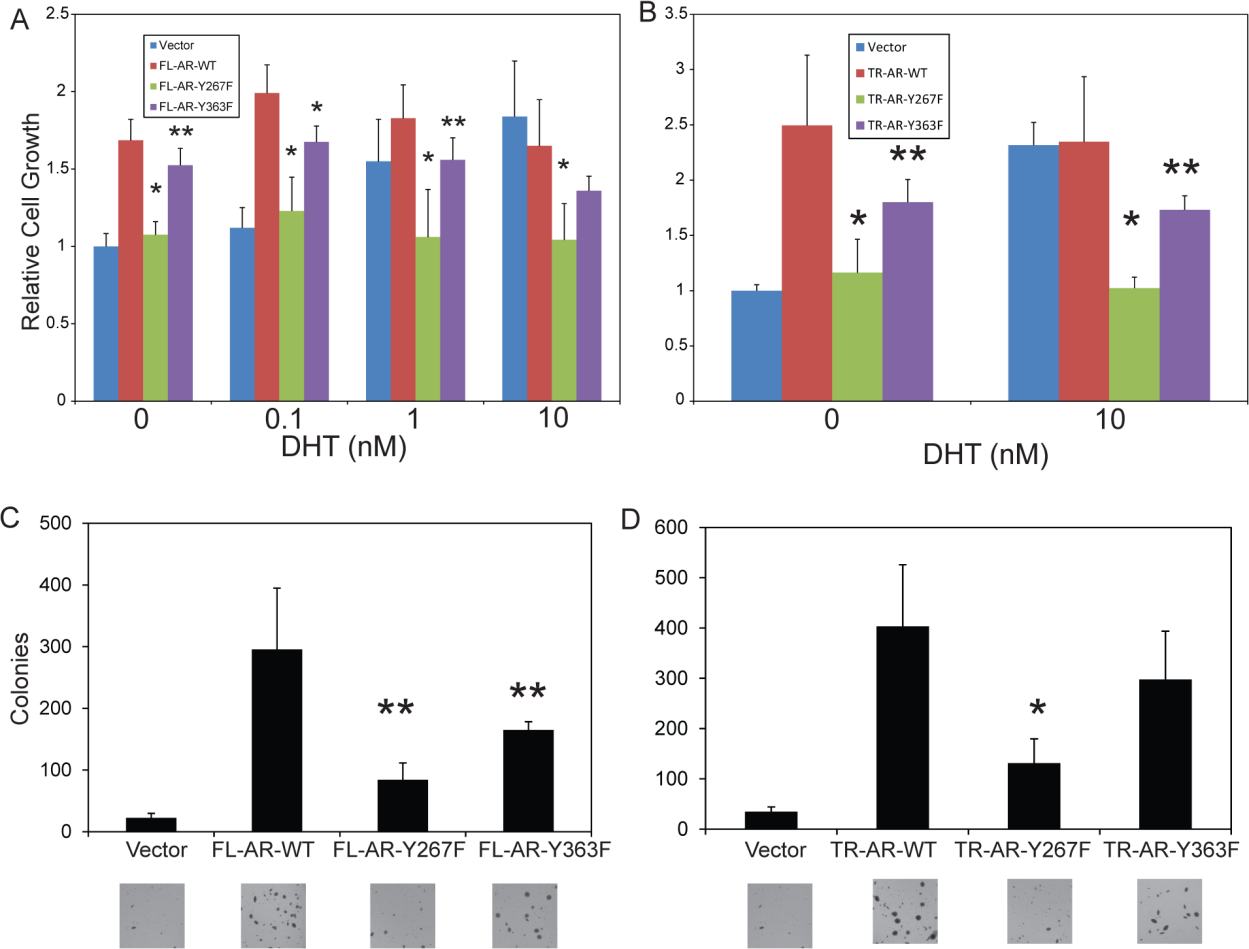
Phosphorylation site mutants of AR are defective in stimulating cell proliferation and anchorage-independent soft agar colony growth. (A, B) LNCaP cells stably overexpressing full length AR (A) or truncated AR (B) were grown in a 96-well plate for 72 hours in media with charcoal-stripped serum and 0, 0.1, 1, or 10 nM of DHT, as indicated. Absorbance at 450 nm was measured after incubation with the colorimetric dye WST-8 for 4 hours. Values were normalized to vector control cells treated with 0 nM DHT. Data shown are the mean + standard deviation of three independent experiments, each performed with triplicate or quadruplicate wells. *, p<0.001 vs AR-WT; **, p<0.05 vs AR-WT by two-group t-test adjusted using the Bonferroni method. (C, D) Cells stably overexpressing full length AR (C) or truncated AR (D) were plated in soft agar and incubated for 3 weeks. Colonies were stained and counted. A representative view of the colonies is shown below. Data shown are the mean + standard deviation of two (C) or three (D) independent experiments with triplicate samples. *, p<0.001 vs AR-WT; **, p<0.05 vs AR-WT by two-group t-test adjusted using the Bonferroni method.

The effect of AR overexpression on cell proliferation at various androgen concentrations was characterized. Proliferation of vector control cells was androgen-dependent and required the full dose (10 nM) of DHT for optimal proliferation (Fig 4A). Full length AR wt overexpressing cells exhibited increased proliferation in the absence of androgen and maximal proliferation at 0.1 nM DHT, a concentration that did not stimulate proliferation of vector control cells. However, Y267F mutant AR-expressing cells did not show increased proliferation in the absence of androgen and did not respond to increasing concentrations of androgen. Proliferation of Y363F mutant AR-expressing cells was intermediate between vector control and AR wt cells in the absence of androgen or at 0.1 nM DHT; the cells did not respond to increasing androgen concentrations. Cells expressing constitutively active truncated AR proliferated optimally without androgen (Fig 4B) and addition of DHT did not increase proliferation. However, cells expressing the truncated AR with the Y267F mutation did not show androgen-independent proliferation and did not respond to androgen treatment. Cells expressing the truncated AR with the Y363F mutation showed modest reduction in androgen-independent proliferation, compared to AR wt and androgen treatment did not increase its growth. The effect of AR overexpression and the effect of phosphorylation site mutation on anchorage-independent growth in soft agar were determined for both full length and truncated AR. Overexpression of full length or truncated AR wt significantly increased soft agar colony formation, compared to vector control cells (Fig 4C and 4D). Overexpression of the Y267F mutant of full length AR or truncated AR demonstrated marked reduction in soft agar colony formation, compared to AR wt. The Y363F AR-expressing showed moderately decreased soft agar colonies, but not to the extent that the Y267F mutant did. These results suggest that the ability of AR to enhance cell proliferation at low androgen concentrations and stimulate anchorage-independent growth in LNCaP cells requires intact phosphorylation sites. Mutation of Tyr-267 exhibits more severe impairment than Tyr-363 in these assays. Collectively, these data suggest that the Tyr-267 and Tyr-363 sites are both required for the full cellular effect of overexpressed AR protein.

### The Y267F mutant of AR is defective in nuclear translocation

**Fig 5:**
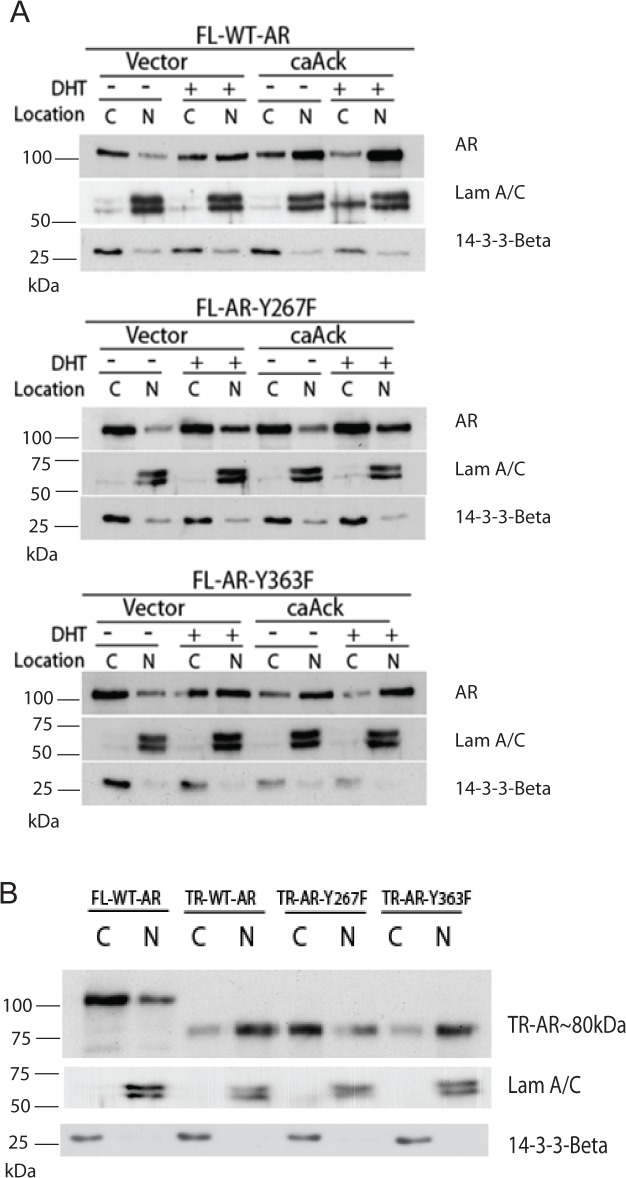
The AR Y267F mutant is defective in androgen- and Ack1-induced nuclear translocation. (A) COS-7 cells were transfected with the AR expression vector and the constitutively active Ack1 L487F expression vector [22] and incubated for 48 hrs. The cells were serum-starved overnight and treated with or without DHT (10 nM) for 2 hrs. Subcellular fractionation was performed. Fifteen μgrams of protein from cytoplasmic (C) and nuclear (N) fractions were immunoblotted with the AR antibody. Laminin A/C and 14-3-3 β were used as markers of nuclear and cytoplasmic fractions, respectively. (B) COS-7 cells were transfected with the AR expression vector (full length or truncated) and incubated for 48 hrs. Subcellular fractionation and immunoblotting were performed as described above.

**Fig 6:**
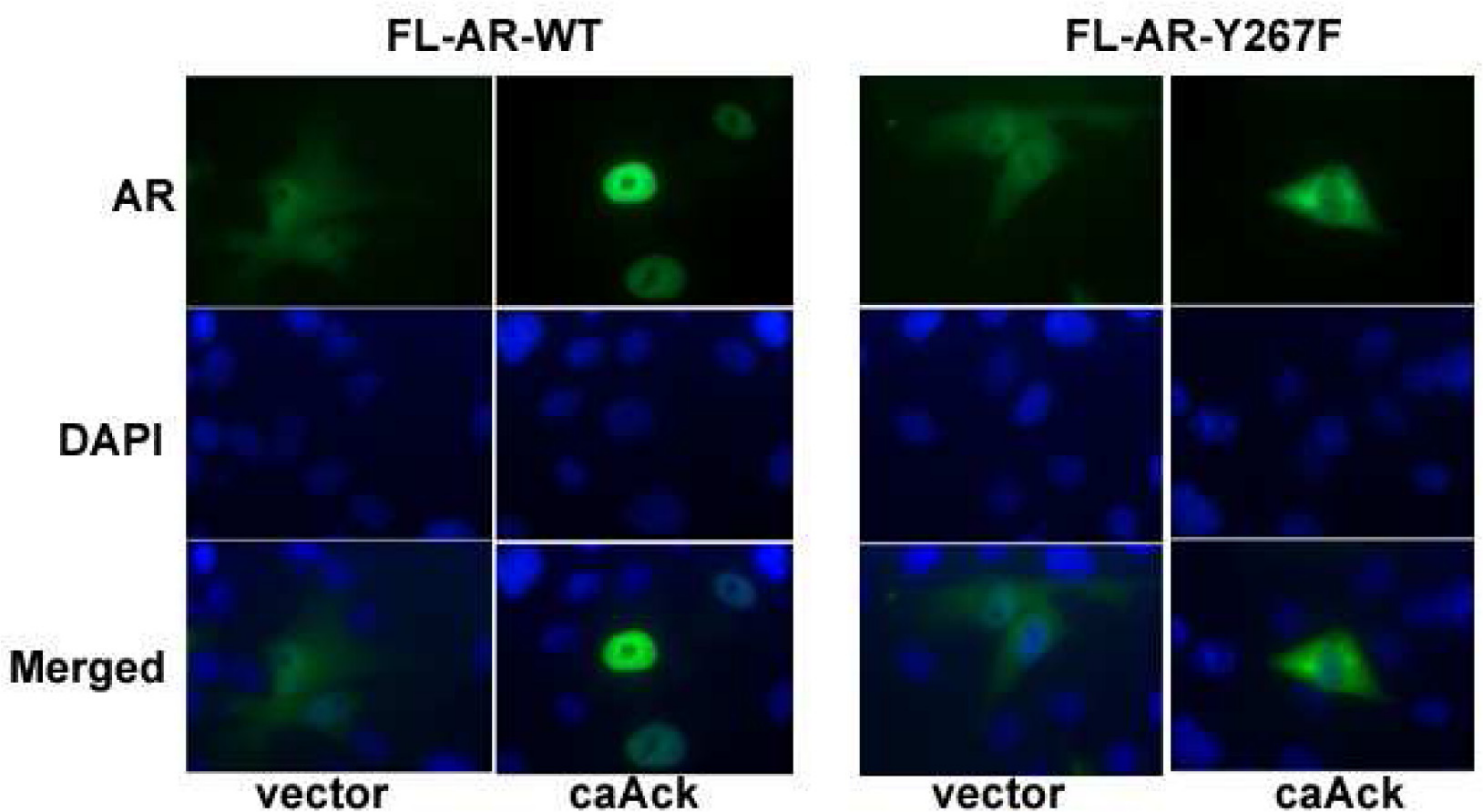
Nuclear translocation of AR induced by Ack1 kinase is defective in the AR Y267F mutant. COS-7 cells were transfected with the AR expression vector and the constitutively active Ack1 expression vector and incubated for 48 hrs. Cells were fixed and AR expression detected with the AR monoclonal antibody and FITC-conjugated secondary antibody. Nuclear staining was performed with DAPI. Original magnifications, 1,000X.

Binding of AR to its ligand causes translocation into the nucleus and binding to the promoter and enhancer regions of its target genes. A previous report indicated that AR nuclear translocation was induced by phosphorylation of AR at Tyr-534 site by Src tyrosine kinase [16]. The effect of Ack1 kinase expression without or with DHT treatment on subcellular localization of AR in COS-7 cells was investigated. Cytoplasmic and nuclear proteins were isolated and probed with an AR antibody. Without DHT treatment, transfected full length AR wt localized mostly in the cytoplasm in cells co-transfected with an empty vector control. However, Ack1 co-expression by transfection increased the amount of AR protein in the nuclear fraction (Fig 5A). In both vector and Ack1 co-transfected with AR wt, there was nuclear translocation in response to DHT treatment. However, DHT treatment or Ack1 co-expression or both DHT-Ack1 together did not induce the nuclear translocation of the AR Y267F mutant to the same extent as AR wt. The nuclear localization pattern of FL-AR-Y363F was similar to AR wt. Immunofluorescence staining of transfected COS-7 cells for AR confirmed that Ack1-induced nuclear localization of AR was inhibited by mutating AR Tyr-267 (Fig 6). These data suggest that AR Tyr-267 is essential for Ack1-induced AR nuclear translocation. Next, the effect of phosphorylation sites on the nuclear localization of truncated AR was evaluated in COS-7 cells. Truncated AR wt in the absence of DHT was localized in the nucleus to a greater extent than full length AR (Fig 5B), as previously reported [29]. The nuclear localization of truncated AR-Y267F was decreased in comparison to wt and the AR Y363F mutant was similar to wt (Fig 5B).

**Fig 7:**
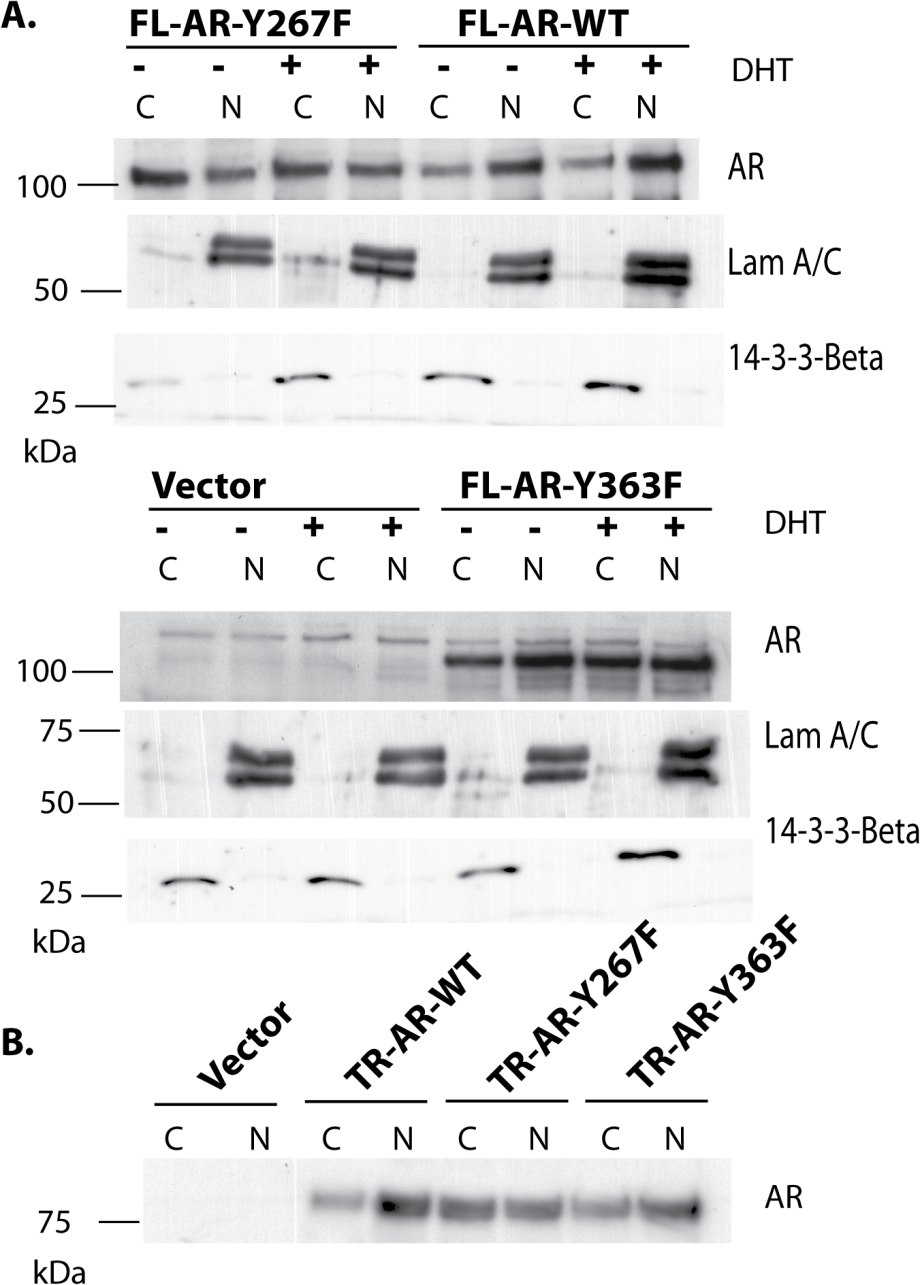
The AR Y267F mutant is defective in nuclear localization in LNCaP cells. (A) LNCaP cells stably overexpressing vector or AR constructs were grown in phenol red free media and serum starved for overnight and treated with or without 10 nM DHT for 2 hrs. Cell fractionation was performed. The cytoplasmic and nuclear lysates from LNCaP cells expressing full length AR constructs were subjected to immunoprecipitation with FLAG antibody, followed by immunoblotting with AR antibody, as indicated. Laminin A/C and 14-3-3 β were used as markers of nuclear and cytoplasmic fractions, respectively. (B) The cytoplasmic and nuclear lysates from LNCaP cells stably expressing truncated AR constructs under androgen-deprived conditions were subjected to immunoprecipitation with FLAG antibody, followed by immunoblotting with AR antibody, as indicated. Laminin A/C and 14-3-3 β were used as markers of nuclear and cytoplasmic fractions, respectively.

In order to confirm the role of Tyr-267 in cells that express endogenous AR, we investigated subcellular localization of exogenous AR protein stably overexpressed in LNCaP cells by immunoprecipitation with the FLAG epitope antibody and immunoblotting with an AR antibody. Overexpression of both full length and truncated AR wt induced predominantly nuclear localization of AR even without DHT treatment (Fig 7A and 7B). The extent of nuclear localization of the Y267F mutant of AR was decreased, compared to AR wt. The Y363F mutant of AR exhibited a pattern of nuclear localization similar to AR wt.

### Y267F mutation inhibits recruitment and DNA binding of truncated AR to the androgen responsive enhancers and transcription of target genes

**Fig 8:**
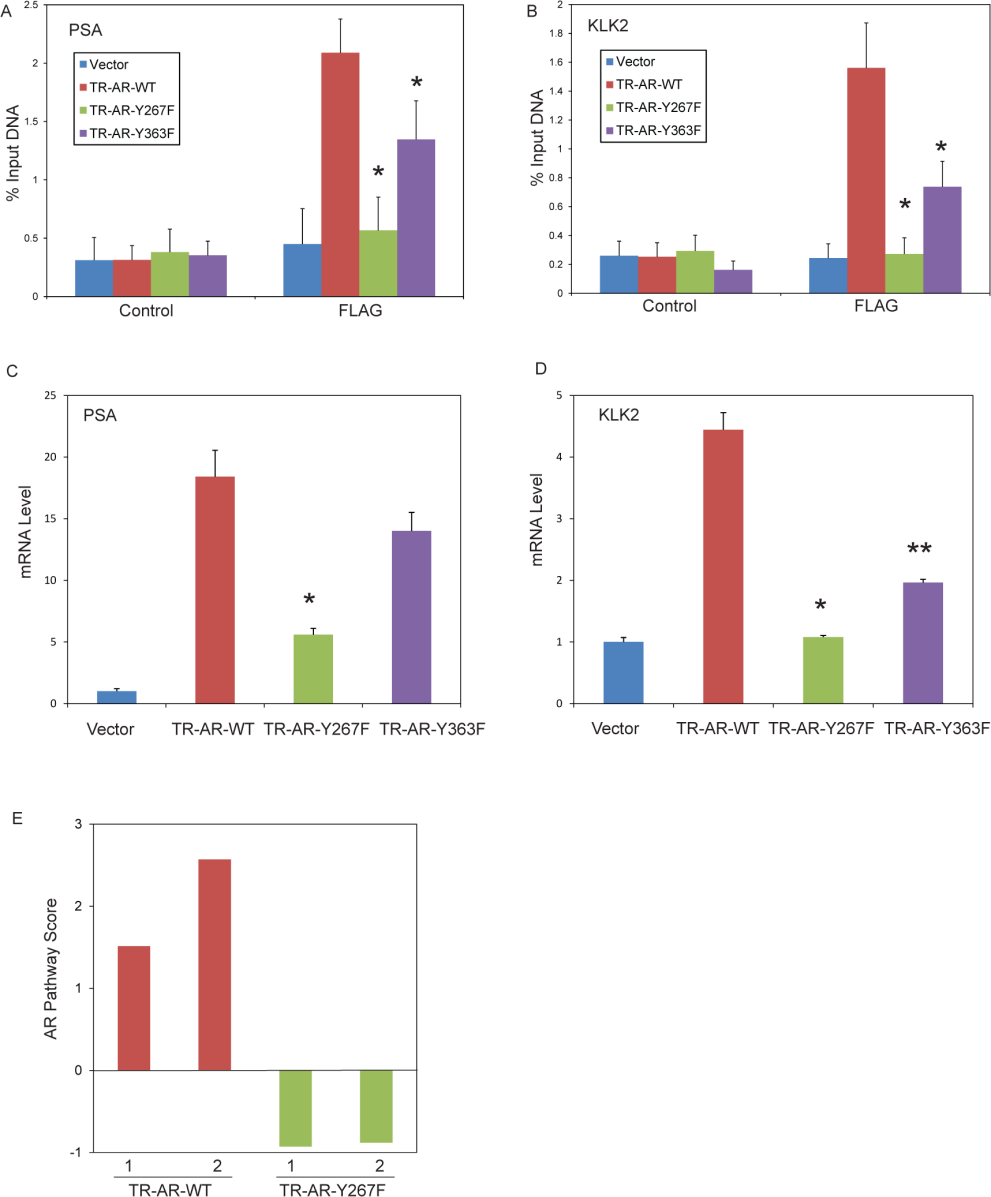
Constitutive recruitment to the chromatin of truncated AR protein and transcription of AR responsive genes are impaired by the Y267F mutation. (A, B) LNCaP cells expressing truncated AR constructs were grown in phenol red free media supplemented with charcoal-stripped serum. Chromatin immunoprecipitation analysis for binding of FLAG-tagged truncated AR protein to the androgen response element III enhancer region of PSA (A) and KLK2 (B) genes were performed. The amount of precipitated DNA was determined by quantitative PCR. The data shown are the mean and standard deviation of three independent experiments performed in triplicate. *, p<0.001 vs TR-AR-WT by two-group t-test adjusted using the Bonferroni method. (C, D) mRNA levels of PSA and KLK2 from LNCaP cells expressing truncated AR constructs under androgen-deprived conditions as above were determined by quantitative RT-PCR performed in triplicate. The data shown are representative of two independent experiments. *, p<0.001 vs TR-AR-WT; **, p<0.05 vs TR-AR-WT by two-group t-test adjusted using the Bonferroni method. (E) Microarray gene expression analysis from LNCaP cells expressing truncated AR constructs under androgen-deprived conditions using vector control as reference was performed. The AR transcriptional pathway activity score was calculated using an established androgen response signature [27]. The score from two independent samples is shown. A positive score indicates increased activation of the AR transcription pathway while a negative score indicates decreased activation of the pathway.

To characterize the effect of Y267F and Y363F mutations on recruitment and binding of AR to the regulatory sequences of target genes, chromatin immunoprecipitation analysis for AR binding to the enhancers of the canonical AR target genes, PSA and KLK2, was performed using the FLAG antibody for detecting the ectopically expressed AR. Truncated AR wt protein was constitutively bound to the PSA and KLK2 enhancers in the absence of androgen. The Y267F mutant did not bind to the PSA and KLK2 enhancers (Fig 8A and 8B). The Y363F mutant also showed decreased binding although the reduction is smaller as that of Y267F. In cells expressing truncated AR wt, PSA and KLK2 mRNA levels measured by quantitative RT-PCR were elevated in the androgen deprived media. However, PSA and KLK2 mRNA levels were decreased in cells expressing truncated AR-Y267F in comparison to AR-wt and to a less extent, in cells expressing truncated AR-Y363F (Fig 8C and 8D). In addition to these two canonical AR target genes, expression of AR responsive genes in these cells under androgen deprived conditions was globally assessed by microarray gene expression analysis. The score representing the AR transcriptional pathway activity was calculated using a published androgen response signature [27]. The AR pathway score was increased in cells expressing truncated AR wt and decreased in cells expressing truncated AR-Y267F (Fig 8E). Together, these results suggested that mutation of the Tyr-267 site diminishes the recruitment and DNA binding of constitutively active truncated AR and transcription of AR responsive genes.

## Discussion

In this study, the potential functional significance of the N-terminal phosphorylation sites Tyr-267 and Tyr-363 in ligand-dependent and—independent activation of AR was investigated by characterizing the effect of tyrosine to phenylalanine substitution mutants in the context of full length and truncated AR. Expression of wild type full length and truncated AR led to increased cell proliferation in the androgen-depleted condition and increased soft agar colony formation. However, the Y267F mutant of full length and truncated AR was defective in stimulating cell proliferation in both androgen-depleted and androgen-supplemented conditions. The Y363F mutant was less severely affected than the Y267F mutant. The full length AR Y267F mutant was defective in nuclear translocation induced by androgen or Ack1 kinase activity. The truncated AR protein used in this study translocated to the nucleus and bound to the androgen enhancers of target genes in the androgen-depleted condition. However, the truncated Y267F AR mutant protein did not exhibit constitutive nuclear localization and enhancer binding activity. Similar to full length AR [24], the truncated AR protein was phosphorylated at Tyr-267 by growth factor-induced activation of intracellular tyrosine kinases (Fig 3). In the previous report, Ack1-induced phosphorylation of AR at Tyr-267 was linked to activation of full length AR and xenograft tumor growth in castrated animals; expression of the full length AR Y267F mutant inhibited Ack1-driven, castrate resistant xenograft tumor growth [23]. In this study, the requirement for the Tyr-267 site even in the constitutively active truncated AR protein, which does not require androgen, has been demonstrated. Inhibition of nuclear translocation, binding to the androgen enhancers, and stimulating cell proliferation in the androgen-depleted condition was observed when the truncated AR Y267F mutant was expressed. Additionally, cells expressing the Y267F mutant of both full length and truncated AR do not exhibit androgen-stimulated cell proliferation. This finding is consistent with the notion that Y267F functions as a “dominant negative” mutant. Ligand-activated AR protein binds to the DNA as a homodimer [31]. Binding between full length AR protein and the truncated AR splice variant protein has been reported [12,32]. Therefore, it may be hypothesized that a complex of endogenous wild type AR and the exogenous Y267F mutant AR is nonfunctional and inhibits the normal cellular response to androgen stimulation. In a similar manner, expression of the ARΔ142–337 transgene with amino acids 142–337 deleted also functioned as a dominant negative mutant in CRPC cells; this construct inhibited CWR-R1 tumor growth [33]. The report by Titus et al and the current data, taken together, support the concept that the AR domain surrounding the Tyr-267 site and phosphorylation of Tyr-267 are required for AR transactivation function, although the mechanisms involved in the dominant negative activity of ARΔ142–337 and the Y267F point mutation may differ.

Since the N-terminal transactivation domain of the AR protein is unstructured and disordered [34], the detailed three-dimensional structure of this AR region has not been determined. Therefore, the effect of Tyr-267 phosphorylation or the Y267F mutation on conformation of the AR molecule is unknown. The AR protein at this residue and other phosphorylation sites likely undergoes rapid cycles of phosphorylation and dephosphorylation and this transient phosphorylation, below the limits of detection by conventional techniques, may be required for optimal AR function. The interaction with components of nuclear import and export machinery as well as coactivators and the basal transcriptional complex may be regulated by phosphorylation at Tyr-267 and potentially Tyr-363. The finding that AR phosphorylated at Tyr-267 may be recruited to enhancers that are distinct from enhancers bound by androgen ligand-activated AR is consistent with this idea that phosphorylation of AR at Tyr-267 adds an additional level of regulation for AR activity. AR phosphorylated at Tyr-267 has been shown to be recruited to the upstream regulatory region of the *ATM* gene and enhance its expression [35]. Sites such as Tyr-534, targeted by Src and Etk/BMX kinases, and some serine/threonine sites (i.e. Ser-81 and Ser-650) may be involved as well. The AR protein phosphorylated by Src at Tyr-534 localizes to the nucleus in androgen-depleted conditions and the phenotype of the Y534F mutant of full length AR resembles that of the Y267F mutant of full length AR described in the current study [16]. Ser-81 of AR is phosphorylated by cyclin-dependent kinase 1 and 9 [36,37]. Chen et al recently demonstrated through expression of the S81A mutant in LNCaP cells that Ser-81 phosphorylation is required for ligand-induced AR recruitment to the androgen enhancers and transcription of target genes [38]. The ligand-activated S81A mutant was localized predominantly in the cytoplasm and chromatin immunoprecipitation analysis showed decreased binding of the S81A AR to the androgen enhancers. The AR bound to chromatin was shown to be phosphorylated on Ser-81. Another group showed that the S81A AR mutant inhibited LNCaP cell proliferation and considered the S81A mutant to exhibit dominant negative activity [39]. The published phenotype of the S81A mutant parallels that of the Y267F mutant described in this study. The stress kinases JNK and p38 regulate the nuclear export of AR by phosphorylation of AR at Ser-650 [40]. Protein phosphatase 1 (PP1) associates with AR and regulates AR transcriptional activity and nuclear localization through dephosphorylation of Ser-650 [41]. Interestingly, a germline mutation S650G of AR results in the mild androgen insensitivity syndrome associated with male infertility and moderately reduced reporter transactivation *in vitro* [42]. This incomplete loss of function of the S650G mutant may stem from loss of phosphorylation at this site. In addition to phosphorylation, other post-translational modifications, such as acetylation, ubiquitination, and SUMOylation, regulate AR function; there may well be crosstalk between phosphorylation and these other modifications [15,43].

There has been much interest in the AR splice variants expressed in tumor cells that are similar in structure to the truncated AR used in this study. These AR splice variants may play a role in progression to castrate resistance and acquired resistance to new therapeutic agents. AR splice variants retain the N-terminal transactivation domain and the DNA binding domain but lack the ligand-binding domain [9–12]. However, it should be noted that the truncated AR 1–660 used in this study differs from naturally occurring AR splice variants in several aspects. The AR 1–660 construct contains most of the hinge region encoded in exon 4 and therefore, the bipartite nuclear localizing signal (NLS) remains intact. However, most of AR variants do not contain exon 4 and the complete bipartite NLS [44]. AR^v567es^, a major splice variant commonly expressed in clinical tumor specimens, does contains the bipartite NLS [12]. The C-terminus of AR splice variants differs from the AR 1–660 construct by the addition of short variable peptide sequences [44]. Presently, the contribution of these C-terminal sequences on the function of AR splice variants has not been elucidated yet. Because of these structural differences between the AR 1–660 and AR splice variants, the requirement for Tyr-267 phosphorylation in naturally occurring AR splice variants requires further investigation.

Two recently approved prostate cancer drugs abiraterone and enzalutamide target the ligand-binding domain of AR by decreasing the ligand availability or inhibiting binding of the ligand. These agents extend the overall survival of CRPC patients by approximately four or five months [45,46]. While these effects are substantial, most patients develop resistance to these drugs and die from progressive prostate cancer. The rise in the PSA level, which may signal AR reactivation in tumor cells, occurs after a median of 8–10 months of treatment. Data from preclinical model systems suggest that emergence of AR splice variants lacking the ligand-binding domain may mediate resistance to enzalutamide and abiraterone [13,14]. Therefore, a new class of drugs is needed to target AR variants without the ligand-binding domain. The finding that loss of phosphorylation sites in the N-terminal domain of AR leads to inhibition of AR activity provides a rationale for development of novel approaches to inhibit AR splice variants.
